# Prophylactic Impella CP versus VA-ECMO in Patients Undergoing Complex High-Risk Indicated PCI

**DOI:** 10.1155/2022/8167011

**Published:** 2022-11-07

**Authors:** Deborah M. F. van den Buijs, Adriaan Wilgenhof, Paul Knaapen, Carlo Zivelonghi, Tom Meijers, Paul Vermeersch, Fatih Arslan, Niels Verouden, Alex Nap, Krischan Sjauw, Floris S. van den Brink

**Affiliations:** ^1^Amsterdam Universitair Medisch Centrum, Vrije Universiteit Medisch Centrum, Amsterdam, Netherlands; ^2^Ziekenhuis Oost-Limburg, Genk, Belgium; ^3^Ziekenhuis Netwerk Antwerpen Middelheim, Antwerpen, Belgium; ^4^Isala Ziekenhuis, Zwolle, Netherlands; ^5^Vivantes Netzwerk für Gesundheit GmbH, Klinikum am Urban Berlin, Germany (present Leids Universitair Medisch Centrum), Leiden, Netherlands; ^6^Medisch Centrum Leeuwarden, Leeuwarden, Netherlands; ^7^Leids Universitair Medisch Centrum, Leiden, Netherlands

## Abstract

**Objectives:**

To compare two different forms of mechanical circulatory support (MCS) in patients with complex high-risk indicated PCI (CHIP): the Impella CP system and veno-arterial extracorporeal membrane oxygenation (VA-ECMO).

**Background:**

To prevent hemodynamic instability in CHIP, various MCS systems are available. However, comparable data on different forms of MCS are not at hand.

**Methods:**

In this multicenter observational study, we retrospectively evaluated all CHIP procedures with the support of an Impella CP or VA-ECMO, who were declined surgery by the heart team. Major adverse cardiac events (MACE), mortality at discharge, and 30-day mortality were evaluated.

**Results:**

A total of 41 patients were included, of which 27 patients were supported with Impella CP and 14 patients with VA-ECMO. Baseline characteristics were well-balanced in both groups. No significant difference in periprocedural hemodynamic instability was observed between both groups (3.7% vs. 14.3%; *p* = 0.22). The composite outcome of MACE showed no significant difference (30.7% vs. 21.4%; *p* = 0.59). Bleeding complications were higher in the Impella CP group, but showed no significant difference (22.2% vs. 7.1%; *p* = 0.22) and occurred more at the non-Impella access site. In-hospital mortality was 7.4% in the Impella CP group versus 14.3% in the VA-ECMO group and showed no significant difference (*p* = 0.48). 30-Day mortality showed no significant difference (7.4% vs. 21.4%; *p* = 0.09).

**Conclusions:**

In patients with CHIP, there were no significant differences in hemodynamic instability and overall MACE between VA-ECMO or Impella CP device as mechanical circulatory support. Based on this study, the choice of either VA-ECMO or Impella CP does not alter the outcome.

## 1. Introduction

In patients with complex coronary artery disease (CAD) or one or more chronic total occlusions (CTO) combined with the presence of comorbidities, determining the optimal revascularization strategy (percutaneous coronary intervention (PCI) or coronary artery bypass grafting (CABG) remains a challenge. These high-risk patients pose an extreme surgical risk and are frequently assigned to medical therapy instead of CABG. However, with the development of new interventional techniques and materials, PCI is a good alternative to CABG and is referred to as complex high-risk indicated PCI (CHIP).

During CHIP, hemodynamics can deteriorate because of temporary complete coronary occlusion or profound myocardial ischemia. This could result in loss of cardiac output and flattening of the arterial curve once the ventricle “uncouples” from the aortic pressure with a risk for cardiogenic shock [1]. Mechanical support during CHIP facilitates native cardiac function by achieving a stable hemodynamic state to withstand repetitive derangements such as ischemia caused by prolonged and repeated balloon inflations, and resume original cardiac function immediately postprocedure or shortly thereafter [2].

There are several mechanical circulatory support (MCS) systems available, i.e., intra-aortic balloon counterpulsation (IABP), Impella, TandemHeart, and veno-arterial extracorporeal membrane oxygenation (VA-ECMO). These MCS have been widely studied in patients with acute myocardial infarction (MI) complicated by cardiogenic shock and showed conflicting results [3–8]. The most important purpose of MCS is to provide an adequate level of hemodynamic support by augmenting mean arterial pressure and cardiac output and thereby avoiding the use of supplementary vasopressor or inotropic therapy.

However, studies regarding the use of MCS in the setting of CHIP are much less abundant and to our knowledge, no study has compared Impella CP with VA-ECMO in CHIP patients [9–11]. This retrospective observational study aims to evaluate all patients who were declined for surgery by the heart team and underwent high-risk PCI with the support of either Impella CP or VA-ECMO.

## 2. Materials and Methods

A multicenter comparative observational study was performed including all patients undergoing CHIP with the mechanical circulatory support of either Impella CP or VA-ECMO.

In patients who underwent Impella CP support, an Impella catheter was inserted through the femoral artery by using a modified Seldinger technique and forwarded into the left ventricle under fluoroscopic guidance. Closure of the Impella femoral access site was performed with a minimum of two Perclose ProGlide suture systems (Abbott Vascular, Redwood City, California). Cannulation of the VA-ECMO was performed using open cutdown and decannulation was accomplished through surgical vascular repair of the femoral artery.

All patients were discussed in the heart team prior to intervention and complex high-risk PCI was defined as patients with an unprotected left main artery, last patent vessel, or complex 3-vessel coronary artery disease, with a left ventricular ejection fraction (LVEF) of ≤30%–40% and severe comorbidities (e.g., severe valve disease(s), renal, pulmonary, or cerebrovascular) that were declined for CABG.

Baseline characteristics were collected including age, gender, prior coronary artery disease, prior CABG, diabetes, history of smoking, hypercholesterolemia, hypertension, prior malignant disease, peripheral artery disease (PAD), renal function, and LVEF. Renal function was defined using the Kidney Disease: Improving Global Outcomes (KDIGO) 2012 Clinical Practice Guideline for the Evaluation and Management of Chronic Kidney Disease (CKD). Left ventricular function was divided into four categories normal (LVEF >55%), mildly impaired (LVEF 45–55%), moderately impaired (LVEF 35–44%), and severely impaired (LVEF < 35%).

The coronary anatomy of both groups was assessed in regard to the target vessel for revascularization, multivessel disease, and the presence of a CTO lesion. For all individual patients, the SYNTAX score and SYNTAX 2 score were calculated using the online Syntax-score calculator (https://syntaxscore.org). The euroSCORE I and II were also calculated for all patients using the online calculator (https://www.euroscore.org/). If there was a CTO lesion present the Japanese-CTO score was calculated using the available online J-CTO calculator (https://www.progresscto.org/cto-scores).

The outcome was analyzed in regard to the successful revascularization of all planned target vessels.

Hemodynamic instability during MCS support was defined as a systolic blood pressure of < 90 mmHg for ≥ 30 minutes or ventricular arrhythmia. If hemodynamic instability occurred, it was resolved with either fluid resuscitation and/or intravenous inotropic or vasopressor agents.

Observed mortality was analyzed in regard to periprocedural mortality, mortality at discharge, and mortality at 30 days. Major adverse cardiovascular events (MACE) were analyzed for both groups.

MACE at 30 days was defined as a composite of all-cause death, myocardial infarction, stroke or transient ischemic attack (TIA), major bleeding events according to the Bleeding Academic Research Consortium (BARC) criteria, repeat revascularization, need for cardiac surgery, limb ischemia, cardiopulmonary resuscitation or acute renal insufficiency. Limb ischemia was assessed by palpation of either the posterior tibial or dorsalis pedis artery of the ipsilateral cannulated femoral artery during the procedure and the occurrence of limb ischemia postprocedure.

Major bleeding events postprocedure were defined according to the BARC criteria and events with a BARC 3 or higher were considered significant and included in the analysis [12].

Deterioration of renal function postprocedurally was the increase of one or more KDIGO stages above baseline. Blood hemoglobin levels in mmol/l were assessed prior to and after the procedure as well as thrombocyte count in 10^6^ units per ml. Transfer to the Intensive Care Unit (ICU) and the mean stay in the ICU as well as on the ward were also analyzed.

Statistical analysis was performed using a Kolmogorov–Smirnov test to assess the normal distribution of continuous data. If there was a normal distribution of the data a Student's *t*-test was performed otherwise a Mann–Whitney *U* test was performed. Categorial variables were analyzed using a Chi-Square test. A two-tailed *p* value of < 0.05 was considered statistically significant.

The data used in this article are derived from previously published data [11, 13]. Both registries were approved by the Medical Ethical Committee at each participating center and written informed consent was obtained from all patients.

## 3. Results

Between 2017 and 2020, a total of 41 patients underwent a complex high-risk PCI facilitated by mechanical circulatory support: 27 patients received support with the Impella CP device (between March 2018 and October 2020) and 14 patients received VA-ECMO support (between January 2017 and July 2018). The decision for the use of mechanical circulatory support was made by the heart team.

The mean age (*p* = 0.44) and the number of men (*p* = 0.15) did not differ significantly between the groups. Other baseline characteristics were well-balanced in both groups (Table 1). Most patients had an LVEF < 35% (70.4% vs. 71.4%). In both groups, 6 patients were known with peripheral artery disease, but either already treated or not significant.

A left anterior descending(LAD) lesion was significantly more apparent in the Impella group (88.9% vs. 57%; *p* = 0.02), but other lesions showed no difference. A majority of the patients in both groups had a CTO (74.1% vs. 71.4%). EuroSCORE, SYNTAX, and J-CTO scores between the two groups did not differ (Table 2), however, a higher J-CTO score occurred in the Impella group (2 vs. 1).

Hemodynamic instability during the procedure occurred in one patient in the Impella group (3.7%) and in two patients in the VA-ECMO group (14.3%). This difference was not statistically significant (*p* = 0.22). All hemodynamic unstable patients developed hypotension with a systolic blood pressure < 90 mmHg for more than 30 minutes and this hemodynamic instability was treated successfully with the administration of intravenous vasopressor agents in two patients and the other patient with fluid resuscitation.

The composite outcome of MACE was higher in the Impella-facilitated CHIP group but showed no significant difference (30.7% vs. 21.4%; *p* = 0.59) (Table 3). Bleeding complications (BARC >3 or higher) occurred more often in the Impella group, but showed no statistically significant difference (22.2% vs. 7.1%; *p* = 0.22). The VA-ECMO patient developed a cardiac tamponade which was successfully evacuated by pericardial drainage. The 6 bleeding complications in the Impella groups had femoral access-site-related bleeding of which 2 patients (7.4%) had significant bleeding from the Impella access site and needed acute vascular surgery to restore the defect. The remaining 4 patients (14.8%) had a grade 3A hematoma at the non-Impella access site but absence of active bleeding. [13].

In-hospital mortality was 7.4% in the Impella CP-assisted patients and 14.3% in the VA-ECMO-assisted patients and showed no significant difference (*p*=0.48). Thirty-day mortality was higher in the VA-ECMO group but did not reach statistical significance (7.4% vs. 21.4%; *p*=0.09).

Limb ischemia did not occur in either group periprocedurally.

Revascularization was successful in 25 patients in the Impella group (92.6%) and in 14 patients in the VA-ECMO group (100%) and showed no significant difference (*p*=0.47).

Kidney function deteriorated in 4 patients in the Impella group (14.8%) and 3 patients in the VA-ECMO group (21%).

There was no statistically significant difference in regard to hemoglobin levels post PCI (6.7 vs 6.4; *p*=0.35) between both groups. The mean length of hospital stay showed no significant difference between the groups but was overall longer in the VA-ECMO group (3 vs. 7 days; *p*=0.23). There was a trend towards more transfer to ICU after Impella in comparison with VA-ECMO (18.5% vs 0%; *p*=0.06).

## 4. Discussion

Our study results indicate that in patients with CHIP who underwent either Impella CP or VA-ECMO assisted PCI, no statistically significant difference in the occurrence of periprocedural hemodynamic instability was observed (3.7% vs 14.3%; *p*=0.22). In the 3 patients, where hemodynamic instability during the procedure did occur, the significant drop in blood pressure was resolved with temporary inotropic agents. In comparison with data from the PROTECT II trial, hypotension during support with an Impella 2.5 occurred in 10.2% of the patients whereas with the Impella CP it occurred in only 3.7% [10]. Data on hemodynamic support with VA-ECMO are scarce. One single-center prospective study on 12 patients stated that the procedures were well tolerated and this could be explained by the stable hemodynamic status of patients in absence of cardiogenic shock or cardiac arrest [14].

VA-ECMO is a modification of the cardiopulmonary bypass circuit that provides a continuous, nonpulsatile cardiac output [15]. It is the only MCS that also oxygenates the blood by removing carbon dioxide from and adding oxygen to venous blood via an artificial membrane [16]. VA-ECMO can provide significant hemodynamic support but has the propensity to increase LV afterload and wall stress, which in turn can increase myocardial oxygen consumption and therefore limit any cardioprotective benefit [17, 18]. To prevent these hemodynamic unfavorable effects, VA-ECMO is in long-term support and generally used in combination with other devices for MCS such as Impella or IABP, but not for short-term support as in CHIP [19, 20]. During VA-ECMO support, vasodilators might reduce afterload and LV end-diastolic pressure, while inotropes can increase contractility [21].

The Impella CP (Abiomed Inc., Danvers, Massachusetts) is a micro-axial pump positioned across the aortic valve which aspirates blood from the left ventricle into the ascending aorta [20]. The effect of LV unloading reduces end-diastolic wall stress, improves diastolic compliance, increases aortic and intracoronary pressure and coronary flow velocity reserve, and stimulates a decrease in coronary microvascular resistance [22].

In our study, the choice of MCS was left to the discretion of the operators, local expertise, and available facilities. Not every patient is suitable for all MCS and the choice depends on patient characteristics such as anatomy and procedural characteristics.

In-hospital and 30-day mortality were not different between both groups, but a trend toward higher 30-day mortality was seen with VA-ECMO-assisted PCI. In both groups, 2 patients died during hospitalization. Careful consideration of these patients by several physicians, including the attending intensivist, cardiologist, interventional cardiologist, and anesthesiologist, concluded that all in-hospital deaths were not related to either the PCI itself or the MCS support. All patients were known with end-stage heart failure and several comorbidities and died as a result of multiorgan failure due to persistent cardiogenic shock. In the VA-ECMO group, mortality is up to 30 days extended to 1 patient. The patient who died after discharge died as a result of persistent heart failure.

No difference in MACE or bleeding complications between both groups was observed in our study population. There were nonetheless numerically more bleeding complications in the Impella group, mainly driven by femoral access site bleeding at the non-Impella site. In only 2 patients there was significant bleeding at the access site. A possible solution to reduce the number of access site bleedings of the non-Impella site is to use the single-access technique for Impella [23]. Using the Impella introducer sheath for PCI access decreases the number of access sites and potentially reduces complications related to multiple access sites.

The reason for no access site bleeding complications in the VA-ECMO group might be explained by a controlled insertion and removal of the cannulas. The cannulas of VA-ECMO are inserted and extracted by surgical cutdown and closure of the femoral artery. In comparison, the femoral access site of the Impella in these patients was closed with the ProGlide closure device. Nonetheless, in only 2 patients in the Impella group the bleeding complications were related to bleeding from the Impella access site, so most bleeding complications arose from the non-Impella access site.

Limb ischemia did not occur in either group. This illustrates the safety of short-term support of MCS during CHIP in regard to limb ischemia. However, in this semi-elective setting of CHIP, patients with known significant peripheral artery disease which could be compromised during or after the procedure, would not be selected for Impella or VA-ECMO. One patient developed venous thromboembolism several weeks after VA-ECMO cannulation at the venous cannulation site. This was resolved without further consequences by treating this patient with oral anticoagulation.

This is to our knowledge the first comparative study between Impella and VA-ECMO in the setting of CHIP. A comparison study between Impella and VA-ECMO in patients with cardiogenic shock showed lower in-hospital mortality, fewer complications, decreased hospital costs, and decreased length of stay in patients supported with Impella in comparison to VA-ECMO. Moreover, there was a higher rate of ischemic stroke and vascular complications in the VA-ECMO cohort [24]. Our study does not display these differences in the setting of CHIP and therefore it is justifiable to use either device in CHIP. Keeping in mind that each MCS has its contra-indications for use, such as LV thrombus for Impella CP and severe aortic regurgitation for both MCS.

This is an observational study and therefore no direct comparison between both groups in a randomized fashion was performed. Data was collected retrospectively with all shortcomings of such. The sample size is relatively small and therefore hypothesis-generating, but may pave the way for future randomized studies.

## 5. Conclusions

In this observational study, there was no difference in hemodynamic instability and MACE in CHIP with either VA-ECMO or Impella CP device for mechanical circulatory support. Although statistically not significant, bleeding complications were higher in patients supported with the Impella CP, but these were mainly driven by bleeding complications from the non-Impella access site. Based on this study the choice of either VA-ECMO or Impella CP does not alter the outcome. Future research, preferably in a randomized fashion, is needed to establish the most effective, safe, and financially favorable form of MCS in patients undergoing high-risk PCI.

## Figures and Tables

**Table 1:** Baseline characteristics.

Baseline Characteristics	Impella CP (*n* = 27)	VA-ECMO (*n* = 14)	*p*-value
Male	20 (74.1%)	13 (92.9%)	0.15
Median age (years)	73 (50–88 ± 9.7)	69 (53–83 ± 8.8)	0.44
Prior CAD	10 (37%)	8 (57.1%)	0.22
CABG	3 (11.1%)	5 (35.7%)	0.06
Diabetes	9 (33.2%)	3 (21.4%)	0.43
History of smoking	11 (37.0%)	4 (28.6%)	0.44
Hypercholesterolemia	11 (40.7%)	4 (28.6%)	0.44
Hypertension	16 (59.3%)	8 (57.1%)	0.90
Prior malignant disease	2 (7.4%)	0 (0%)	0.30
Peripheral artery disease	6 (22.2%)	6 (42.8%)	0.17
Impaired renal function (eGFR <60, stage ≥3)	10 (37.0%)	3 (21.4%)	0.31
LVEF > 55%	2 (7.4%)	0 (0%)	0.30
LVEF 45–55%	0 (0%)	1 (7.1%)	0.16
LVEF 35–45%	6 (22.2%)	3 (21.4%)	0.95
LVEF < 35%	19 (70.4%)	10 (71.4%)	0.94

CABG = coronary artery bypass graft; CAD = coronary artery disease; LVEF = Left ventricular ejection fraction.

**Table 2:** Coronary anatomy.

Coronary Anatomy	Impella CP (*n* = 27)	VA-ECMO (*n* = 14)	*p* value
Left main	18 (66.7%)	10 (71.4%)	0.76
Left anterior descending	24 (88.9%)	8 (57.0%)	0.02
Isolated right coronary artery	3 (11.1%)	1 (7.1%)	0.68
Multivessel disease	22 (81.5%)	10 (71.4%)	0.46
Patients with a CTO	20 (74.1%)	10 (71.4%)	0.86
J-CTO score	2 (0–4)	1 (0–3)	0.81
Syntax score	32 (8–57)	34 (8–42.5)	0.98
Syntax 2 score for PCI	51.7 (30.5–80.7)	53.5 (26.2–79.5)	0.76
Syntax 2 score for CABG	40.7 (22.3–64.9)	40.1 (16.2–57.2)	0.82
EuroSCORE (%)	7.25 (1.33–49.66 ± 12.76)	7.1 (3.6–34.1 ± 10.4)	0.94

CABG = coronary artery bypass grafting; CTO = chronic total occlusion; PCI = percutaneous coronary intervention.

**Table 3:** Procedural characteristics and outcome.

Procedural Characteristics and Outcome	Impella CP (*n* = 27)	VA-ECMO (*n* = 14)	*p*-value
Hemodynamic instability	1 (3.7%)	2 (14.3%)	0.22
Periprocedural mortality	0 (0%)	0 (0%)	1.00
Mortality at discharge	2 (7.4%)	2 (14.3%)	0.48
Mortality at 30 days	2 (7.4%)	2 (21.4%)	0.09
MACE at 30 days	10 (37.0%)	4 (21.4%)	0.59
Bleeding complications (BARC ≥3)	6 (22.2%)	1 (7.1%)	0.22
Grade 3A	4 (14.8%)	0 (0%)	0.12
Grade 3B	2 (7.4%)	1 (0%)	0.98
Access site related	2 (7.4%)	0 (0%)	0.30
Limb ischemia	0 (0%)	0 (0%)	1.00
Successful revascularization	25 (92.6%)	14 (100%)	0.47
Renal function postprocedural (increase ≥1 stage above baseline)	4 (14.8%)	3 (21%)	0.80
Hb prior to PCI (mmol/l)	7.7 (5.3–9.9 ± 1.2)	8.3 (6.9–9.8 ± 0.93)	0.13
Hb post PCI (mmol/l)	6.7 (5.2–8.6 ± 1.1)	6.4 (5.1–7.5 ± 0.76)	0.35
Transfer to ICU	5 (18.5%)	0 (0%)	0.06
Mean stay in ICU (days)	1 (1–1 ± 1)	0 (0–0 ± 0)	N/A
Mean stay in hospital (days)	3 (1–23 ± 4.76)	7 (2–28 ± 7.2)	0.23

ICU = intensive care unit; MACE = major adverse cardiovascular events; PCI = percutaneous coronary intervention.

## Data Availability

The data used to support the findings of this study are available from the corresponding author (F.S. van den Brink) upon request.
